# Endovascular Repair of Aortic Dissection in Marfan Syndrome: Current Status and Future Perspectives

**DOI:** 10.3390/diseases3030159

**Published:** 2015-07-27

**Authors:** Rosario Parisi, Gioel Gabrio Secco, Marco Di Eusanio, Rossella Fattori

**Affiliations:** 1 Cardiology and Interventional Cardiology Unit, AO Ospedali Riuniti Marche Nord, Pesaro, Piazzale Cinelli 1, 61121 Pesaro, Italy; E-Mails: r.parisi@ospedalimarchenord.it (R.P.); gioel.gabrio.secco@gmail.com (G.G.S.); 2 Cardiac Surgery Unit, G. Mazzini Hospital, Piazza Italia 1, 64100 Teramo, Italy; E-Mail: marco.dieusanio2@unibo.it

**Keywords:** Marfan Syndrome, aortic dissection, TEVAR

## Abstract

Over the last decades, improvement of medical and surgical therapy has increased life expectancy in Marfan patients. Consequently, the number of such patients requiring secondary interventions on the descending thoracic aorta due to new or residual dissections, and distal aneurysm formation has substantially enlarged. Surgical and endovascular procedures represent two valuable options of treatment, both associated with advantages and drawbacks. The aim of the present manuscript was to review endovascular outcomes in Marfan syndrome and to assess the potential role of Thoracic Endovascular Aortic Repair (TEVAR) in this subset of patients.

## 1. Introduction

Marfan syndrome (MFS) is an autosomal dominant disorder of the connective tissue caused by mutation in the FNB1 gene on chromosome 15, with high penetrance and varying phenotypic expression, which involves the cardiovascular, ocular and muscolo-skeletal systems [1]. The incidence is of 1/10,000 patients with no known gender or ethnic predilection. The gene encodes fibrillin 1 glycoprotein, which is essential for the formation of elastic fibers found in connective tissue, provides a scaffold for elastin deposition in the extracellular matrix, fundamental to maintain the integrity of the vessel wall and serves as a regulator of transforming growth factor beta (TGF-β) signaling [2]. The Ghent criteria, first reported in 1996 and reviewed in 2010 by Loeys *et al.*, are currently utilized to diagnose the Syndrome [3]. Patients with MFS are prone to developing vascular complications such as aortic dilatation, dissection and rupture. The life expectancy of these individuals has increased tremendously, by 30 years, over the past 30 years [4]. This is due to medical management with β-blocker and AT1 antagonists therapy [5,6,7] but especially to improved surgical results of early prophylactic aortic interventions on the proximal thoracic aorta [8]. If in the early 1970s, the mean life expectancy for these patients was 32 ± 16 years, between 1972 and 1995 the median (50%) cumulative probability of survival was 48 to 72 years [8,9].

In 1999, in a survey involving 10 major Marfan centers, Gott and colleagues [10] showed excellent surgical outcomes reporting a 30-day mortality rate of 1.5% for patients undergoing elective aortic root replacement. However, because of the progressive nature of these diseases, even after successful aortic root replacement, the number of MFS patients requiring secondary interventions on the descending thoracic aorta (DTA) or thoraco-abdominal aorta due to new or residual dissections, rupture and distal aneurysm formation has substantially increased [11].

Open repair of the descending aorta in these patients is highly challenging and excellent surgical expertise is important to minimize catastrophic complications such a stroke, paraplegia or paraparesis, postoperative hematomas and death [12]. Higher complication rates have been reported by low-volume centers with fewer than four cases/year, where in-hospital postoperative mortality can be as high as 27%, compared to less than 10% recorded in some high-volume centers [13,14,15].

Thoracic Endovascular Aortic Repair (TEVAR) has emerged as valid alternative to open surgery in the past decades gaining wide acceptance in the treatment of acute and chronic type B aortic dissection, considering the unsatisfactory results of open repair. However, the role of TEVAR in MFS remains a frequently debated issue.

## 2. Literature Review

To date, only small series of MFS patients treated with endovascular approach have been published. Baseline and follow up results of these studies are summarized in Table 1 and Table 2.

Ince [16] and coll. reported a series of six consecutive MFS patients treated with TEVAR. Indications for intervention were chronic type B dissections after previous aortic root repair in five patients and solitary type B dissection in one patient. Talent stent grafts were successfully deployed in all cases, with no 30-day mortality or 1-year procedure-related mortality. Complete aortic remodeling was observed in two cases. At a mean follow up of 51 months three patients required open conversion at 12, 22 and 43 months. One patient with a known dilated aortic root suddenly died after 24 months, and another was reported under surveillance for retrograde false lumen flow without aortic diameter increase.

**Table 1:** Thoracic Endovascular Aortic Repair (TEVAR) in Marfan Syndrome: Preoperative status and early outcomes.

Author	No. of patients	Urgent/Emergent Status (*n*; %)	Previous Aortic Surgery (*n*; %)	In-hospital Mortality (*n*; %)	Paraplegia/Paraparesis (*n*; %)	Stroke/TIA (*n*; %)	Primary Endoleak (*n*; %)
**Ince *et al.* 2005 [16]**	6	NA	5 (83%)	0	0	0	0
**Nordon *et al.* 2009 [17]**	7	3 (42.8%)	7 (100%)	1 (14%)	0	0	NA
**Geisbusch *et al.* 2008 [18]**	6	NA	3 (50%)	0	0	0	*Type I:* 1 (16.6%)
**Botta *et al.* 2009 [19]**	12	5 (41.7%)	12 (100%)	0	0	0	*Type I:* 1 (8.3%)
							*Type II:* 2 (16.7%)
**Marcheix *et al.* 2008 [20]**	15	2 (13.3%)	11 (73%)	0	0	1 (6.7%)	*Type I:* 4 (26.7%)
							*Type II:* 1 (6.7%)
**Waterman *et al.* 2012 [21]**	16	3 (18.7%)	15 (94%)	1 (6.2%)	NA	NA	*Type I:* 3 (18.7%)
							*Type II:* 1 (6.2%)
**Eid-Lidt *et al.* 2013 [22]**	10	10 (100%)	5 (50%)	1 (10%)	0	1 (10%)	NA

NA: Not avaible.

**Table 2:** TEVAR in Marfan Syndrome: Follow up.

Author	No. of patients	Mean Follow up (months)	Secondary Endoleak (*n*; %)	New Endovascular Procedure (*n*)	Surgical Conversion (*n*)	Late Death (*n*)
**Ince *et al.* 2005 [16]**	6	51 (12–74)	NA	0	2	1
**Nordon *et al.* 2009 [17]**	6	16 (3–54)	*Type I:* 1 (16.7%)	2	0	1
			*Type III:* 1 (16.7%)			
**Geisbusch *et al.* 2008 [18]**	6	32.8 (3–79)	*Type I:* 1 (12.5%)	1	0	1
			*Type III:* 1 (12.5%)			
**Botta *et al.* 2009 [19]**	12	31 (3–57)	*Type I:* 1 (8.3%)	1	1	0
			*Type III:* 1 (12.5%)			
**Marcheix *et al.* 2008 [20]**	15	25 (10–59)	*Type I:* 4 (26.7%)	3	5	3
			*Type III:* 1 (6.7%)			
**Waterman *et al.* 2012 [21]**	15	9 (0–46)	NA	4	7	3
**Eid-Lidt *et al.* 2013 [22]**	9	59.6 (9–102)	*Type I:* 1 (22.2%)	3	0	1
			*Type II:* 1 (22.2%)			
**Total**	69	32	13 (18.8%)	14	15	10

NA: Not avaible.

Nordon [17] and coll. reported on seven MFS patients undergoing TEVAR for aneurismal chronic dissection after previous aortic root repair. Technical success was 100%, one patients (14%) died due to pneumonia and irreversible cardiac failure. No perioperative adverse neurologic events occurred. At a median follow of 16 months, two patients developed endoleaks successfully managed within the first year by additional stent graft deployment. However, at imaging follow up, all patients demonstrated continued thoracic aorta dilatation with an alarming average of 7 mm per year.

Geisbusch [18] and colleagues treated 167 patients with TEVAR between 1997 and 2007. Of them, eight patients presented with a diagnosis of connective tissue disease (six Marfan and two Ehler-Danlos). The pathologies included thoracic and thoracoabdominal aortic lesions (one degenerative aneurysm and seven chronic expanding aortic dissections). Technical success was 88% (one primary type one endoleak). There were no operative deaths. At a median follow up of 31 months, seven (88%) were alive. Endoleaks were observed in three patients after endovascular treatment of residual chronic type A dissection. Half of patients showed progression of disease with de novo aneurysms or aortic expansion. No patients needed early or late conversion to open procedures, but three (38%) underwent endovascular re-interventions.

Botta [19] and colleagues investigated the feasibility and outcomes of TEVAR of the descending aorta in 12 MFS patients already submitted to open aortic root/arch surgery. Stent graft procedures were performed urgently in five patients and electively in seven. Neither in-hospital death nor adverse neurologic events occurred. At a median follow up of 31 months, one patient underwent open surgical conversion for persistent type 1 endoleak about 3 months after endovascular repair. Two patients showed extension of the dissection at 1 and 24 months after the procedure. No late death or aortic rupture was observed.

Marcheix [20] and coll. analyzed short and mid-term outcomes of 15 MFS patients with chronic aortic dissection from the 457 enrolled in the European Talent registry. Eleven patients had previously aortic root surgery. Thirteen patients with progressive descending aortic dilatation were treated electively. Two patients were treated urgently due to impending aneurismal rupture. Technical success was achieved in all cases, and no conversion to open surgery was required during the procedure. No deaths or paraplegia occurred. One patient suffered a transient hemispheric stroke. Primary endoleaks were detected in five patients (type 1, *n* = 4; type 2, *n* = 1). The primary type 2 endoleak spontaneously healed. Of the remaining four patients with type 1 primary endoleak, one successfully underwent secondary conversion to open surgery one month after TEVAR, one died after endovascular reintervention, and two died of rupture of the descending aorta or of the abdominal aorta (within post-operative day 335) before scheduled reinterventions. After a mean follow up time 2.1 years, 12 patients were alive. Of them, five successfully underwent secondary conversion to open repair and seven showed different degrees of false lumen thrombosis.

Waterman [21] and coll. examined the outcomes in 16 patients with MFS undergoing TEVAR between 2000 and 2010. Fifteen patients had previous surgery of the ascending aorta or arch. Chronic dissection and/or aneurysmal dilation of the descending aorta were the indications for elective intervention in 13 patients. Two acute dissection/malperfusion cases and one anastomotic disruption early after an open surgery led to emergency intervention in three patients. Six patients (38%) had successful therapy and required no subsequent interventions over a median follow up of 6.8 months. Seven (44%) patients experienced primary treatment failure defined as type 1 endoleak, persistent false lumen flow in the stented aorta leading to aneurysmal degeneration, and/or need of subsequent open or endovascular interventions. Five of these patients required open conversion. Five patients died during follow-up. Of them, two died perioperatively: the patient who underwent emergent TEVAR for anastomotic disruption, and a patient who required multi-visceral revascularization in conjunction with a second TEVAR. Two patients died following discharge: one because of a respiratory failure at 3 months, and the other because of a cardiac arrest after 4 months. The remaining patient died more than 6 years after EVAR from advanced age (84 years).

Eid-Lidt and coll [22] evaluated mid-term follow-up in 10 Marfan patients with acute aortic syndrome complicating a chronic type B dissection (four contained rupture, one malperfusion syndrome and five acute aortic expansion). Five patients had previous surgical procedure. Technical endovascular success was achieved in all patients. In-hospital mortality was 10% (one patient died of aortic rupture 5 days after TEVAR during an open surgical repair). There were no cases of paraplegia or paraparesis. One patient had a transient ischemic attack 2 days after TEVAR without recurrence and one patient had vascular access complications. At a mean follow-up of 59.6 months, the cumulated mortality was of 20% and late mortality 11.1%. The rate of secondary endoleak was 44.4% and late reintervention of 33.3%. Survival freedom from cardiovascular death at 8 years was 80.0% and positive remodeling was documented in 37.5% of patients.

## 3. Discussion

Despite the small number of patients and the heterogeneity of treated diseases (acute and chronic dissection and aneurism), common finding of these studies are the feasibility of endovascular technique in MFS patients with percentage of success nearly to 100% with low morbidity and mortality rates. However, mid term follow up data reveal a high reintervention rate due to primary and secondary endoleaks and reduced rate of positive aortic remodeling. Despite major improvement in interventional materials and techniques, the increase in circumferential stress of the vessel wall created by the radial force of the stent graft might be deleterious in this clinical setting where a particularly fragile aortic wall is usually encountered. Landing a stent graft in the native aorta in patients with connective tissue disease is subjected to early and late complication. In a series reported by Dong *et al.* [23] the most common complication in MFS patients was retrograde aortic dissection (rTAD). This was confirmed in an analysis from the European registry on endovascular aortic repair complications, where the presence of a connective tissue disorder accounted for 8.4% of the underlying disease [24]. Consequently, it is prudent to consider TEVAR in MFS patients only with devices without proximal bare springs, if logistically feasible. Also, in a report of 650 patients treated for type B aortic dissection with endovascular approach, 22 events of stent graft–induced new entry (SINE) tears occurred. The mortality of the new tears was substantial, with nearly 30% of patients dying from the event. The incidence of SINE in the patients with MFS was 33%, which was significantly higher than the 3% among non-Marfan patients [25]. In general, patients with genetically triggered aortic diseases are excluded by the criteria currently approved by the US Food and Drug Administration for using commercially available thoracic endovascular devices to treat aneurysms in the descending thoracic aorta. In addition, the expert consensus document for endovascular therapy in patients with descending thoracic disease does not recommend stent grafts unless conventional repair poses a prohibitive risk [26,27]. More recent in a position statement [28] TEVAR is not recommended in patients with connective tissue disease except as a bail-out procedure or bridge to definitive open surgical therapy, or as a procedure following prior aortic repair when both landing zones lie within previously sited prosthetic grafts. Finally, last European guidelines on diagnosis and treatment of aortic diseases say that, in case of Marfan disease, surgery should be preferred over TEVAR (class IIa, level of evidence C), except in emergency situation in order to get initial stabilization as a bridge to definitive surgical therapy [29]. In summary, TEVAR could be an option in patients with absolute contraindication to open thoracic aorta surgery or, in selected patients, when the graft can be placed within a proximal and distal prosthetic graft landing zone. Certainly, endovascular treatment remains important in the emergency conditions, such as complicated type B acute aortic dissections and ruptured true or false aneurysms for which surgical therapy is associated with sobering outcomes and rapid control of the disease is essential to increase patients’ survival. Finally, close clinical and imaging surveillance is mandatory to detect early stent graft complications and/or disease progression of the native aorta.

## 4. Conclusions

In MFS patients, the management of the aortic pathology is complex and often requires remedial procedures. Open surgery remains the mainstay of the treatment but endovascular therapy can provide a useful adjunct or bridge to open surgical treatment in anatomically suitable patients. However, failure of endovascular therapy is common, and its use should be judicious with close follow-up to avoid delay if open surgical repair is required.
